# The Relationship Between Narrative Medicine and Nurse and Nurse Practitioner Well-Being

**DOI:** 10.3390/nursrep16010032

**Published:** 2026-01-20

**Authors:** Paulette J. Thabault, Emily Gesner

**Affiliations:** 1 School of Nursing, Bouve College of Health Sciences, Northeastern University, Boston, MA 02115, USA; 2 School of Nursing, Norwich University, Northfield, VT 05663, USA; 3 Dartmouth-Hitchcock Medical Center, Lebanon, NH 03756, USA; emily.j.gesner@hitchcock.org

**Keywords:** narrative medicine, nurse well-being, burnout, empathy, nurse practitioner well-being

## Abstract

**Background:** Narrative Medicine (NM) has emerged as a strategy to support reflective clinical practice and emotional resilience among nurses. This study examined relationships between NM practices and well-being among registered nurses (RNs) and nurse practitioners (NPs). **Methods:** A national sample of RNs and NPs was recruited using snowball sampling. Participants completed a NM practice survey and the Mayo Clinic Well-Being Index (WBI) survey. Data were analyzed using descriptive statistics and Pearson correlation coefficients. **Results:** A total of 3167 responses were analyzed (1934 RNs and 1233 NPs). Among RNs, strong statistically significant correlations were found between NM practices and well-being scores (*p* < 0.001). Among NPs, moderate correlations appeared in select NM practice dimensions (*p* < 0.05). **Conclusions:** Engagement in narrative Medicine practices is associated with improved well-being among nurses and nurse practitioners. NM may present a promising strategy to reduce burnout and strengthen professional resilience.

## 1. Introduction

As health care systems across the country continue to face mounting staffing challenges, registered nurses (RNs) and advanced practice nurse practitioners (NPs) are among the top health care practitioners experiencing burnout. In a 2022–2023 State of Well-Being Report, overall, 49% of RNs and 44% of NPs reported feeling distressed or struggling with younger cohorts in both groups reporting higher levels [1]. Feelings of burnout, emotional hardening, depression or hopelessness, daytime sleepiness, a sense of things piling up and emotional problems were measured. Physical health problems and work interfering with daily life and meaningfulness of work were also measured. RNs and NPs at high risk of burnout experienced poorer overall quality of life, reported recent patient care errors, and an intent to leave their current nursing position. Burnout has been characterized by the World Health Organization with three dimensions: (1) feelings of energy depletion or exhaustion, (2) increased mental distance from one’s job, and (3) feelings of negativism related to one’s job and reduced professional efficacy [2].

Burnout among nurses has an alarmingly negative impact on the nursing workforce. In 2021, 100,000 nurses (an overall 3.3% of nurses) left the workforce, citing burnout, pandemic impacts, and the nursing shortage as key factors [3]. Moreover, nursing burnout is associated with poorer outcomes and reduced safety in patient care and contributes to rising nursing turnover [4,5,6,7]. Nearly 18% of newly licensed RNs leave the profession in the 1st year [8,9]. Between 22–32% of the nursing workforce is considering leaving the profession [10], and depending on geographical location, nursing turnover ranges from 8 to 27% and NP turnover is alarming at 10% [11]. Additionally, the US Bureau of Labor Statistics projects that from 2020 to 2030 more than 275,000 additional nurses will be needed. This phenomenon is further exacerbated by declining numbers of nursing faculty and nursing program applications [6,12].

The reasons RNs and NPs cite for leaving the profession are similar and include stressful working conditions, lack of leadership and supervision, and understaffed facilities [13]. Improving the practice environment, valuing a team culture, supporting work–life balance, and providing sufficient resources are among the key strategies that may be needed to reduce burnout [14,15].

It is imperative that the nursing profession find ways to support nurses at all levels to stem the rising rates of burnout and turnover.

Narrative Medicine may provide an avenue for improving RN and NP job satisfaction and for improving the quality of care for our patients. Narrative Medicine is defined as a “practice with the narrative competence to recognize, absorb, interpret and be moved by the stories of illness” and “a clinical practice informed by the theory and practice of reading, writing, telling, and receiving of stories” [16].

Integrating NM into nursing practice supports the formation of trusting relationships with patients, families, and colleagues, promotes nurse resilience, and reduces stress and burnout [17]. Narrative Medicine in nursing practice is a transformative model where nurses at all levels of practice not only observe and respond to objective information about patients and their illnesses but also connect to their patients to understand their fears and hopes and the implications of their illness. NM calls upon us to speak humbly, genuinely, and deeply with patients, families, and other health care professionals [18].

However, NM goes further than listening and understanding the story of another. NM is twofold: to truly connect to our patients’ stories, NM calls for a reflective practice, identifying and interpreting one’s own emotional responses to clinical experiences and making sense of how those experiences influence one’s own personal and professional path. Ultimately, with this deeper understanding, nurses can integrate NM insights into their future nursing practice [17,18].

NM employs deep listening, storytelling, and reflective practices such as journaling, poetry, art, and music to allow clinicians to assess and appreciate clinical experiences. Nurses and nurse leaders participating in one narrative nursing workshop reported feeling empowered, valued, and fortunate to have taken part in the workshops. The structured workshop focused on the value and techniques of storytelling and included small group reflection. The results suggested a positive impact on their professional identities and sense of purpose [19]. D’Silva et al. found the highest levels of job satisfaction and the lowest levels of burnout amongst health care workers who identified with an organizational “clan” culture. “Clan” culture prioritizes employee well-being, cohesiveness, engagement, and teamwork through human affiliation, collaboration, trust, loyalty, and support [20].

NM practices have been integrated into multiple clinical settings, demonstrating not only improved health care provider experience but also improved patient care [19]. Studies have demonstrated improvement in patient symptoms, emotional health, and quality of life with reflective writing, drawing, speaking, and even singing about meaningful experiences. NM activities, including journaling, writing prose or poetry, or graphic medicine alone or in a group setting, are effective practices [19].

Moreover, storytelling, central to NM, has demonstrated support for diverse populations. When researchers introduced healing circles and talking circles into primary care with Native Americans, significant improvement occurred in symptoms, activities of daily living, and overall well-being during the times that participants attended the talking circles [21].

In response to burnout across disciplines, high turnover, and recruitment challenges, NM programs are emerging in education and clinical environments. Programs focus on enhancing opportunities for storytelling, listening to our patients’ stories, building community across professions, and improving the dialogue amongst colleagues and patients [16,22,23,24]. Narrative Medicine promotes professionalism, empathy, and humanistic care in nursing practice [25].

Narrative Medicine relies on essential attentive listening skills, leading to a deeper understanding of our patients’ stories, empathy and compassion for the story of another, and appreciation for how we are impacted by the story of another. The foundational principles of NM are attention—the capacity to listen for the twists and turns of another person’s experiences with intensity and empathy; representation—the ability to create a story that captures that other person’s lived reality; and affiliation—the desire to share a common bond with the other person and be moved to action on their behalf [16,26].

## 2. Research Question

This study investigated the relationship between Narrative Medicine practice and well-being among practicing professional nurses and nurse practitioners. The researchers hypothesized that when nurses and nurse practitioners practice the principles of NM, they experience higher levels of well-being and reduced burnout.

## 3. Materials and Methods

The survey was conducted online using email or social media, such as LinkedIn, to invite nurses and nurse practitioners to participate. Nurses and nurse practitioners were surveyed about their well-being and about their nursing or nurse practitioner practice. Two surveys were utilized. The first survey, developed by the researcher with an expert reviewer, assessed the respondent’s knowledge of NM and the extent to which they incorporated principles of NM into their nursing or NP practice. The survey was designed using Charon’s key principles of Narrative Medicine practice, and the expert reviewer was trained in Narrative Medicine and developed and taught Narrative Medicine at a University. The NM survey consisted of 12 statements that align with NM principles. Using a Likert scale, it asked respondents to rate their level of agreement with the statements, reflecting their beliefs and practices. Three questions asked respondents to rate the level of importance of NM practices of listening to their patients’ stories, having empathy, and mindful listening. Five questions assessed the degree to which the respondent believed the incorporation of certain NM practices (obtaining the patient’s story, learning the context of illness in their patient’s life, recognizing the impact of not hearing the patient’s story, and developing a partnership with their patient), made them better RNs or NPs, improved diagnosis, or improved patient outcomes. Two questions asked respondents to rate their level of engagement or practice with hearing a patient’s story and engaging in reflective practices. One question asked respondents to rate the degree to which hearing a patient’s story increases self-understanding. One question assessed the degree to which the respondent believed developing professional affiliations made their practice more rewarding. The second survey, the Mayo Clinic Well-Being, assessed RN and NP well-being (Supplementary Materials: Mayo Clinic Well-Being Measurement Surveys for Registered Nurses and Advanced Practice Providers).

This study was approved by the Institutional Review Board of the University where one of the researchers was employed. Participants were recruited from a national sample using a snowball sampling method. Inclusion criteria were registered nurses and nurse practitioners employed in clinical practice. The exclusion criteria were nurses who do not work in a clinical setting or health care workers who do not hold a registered nurse license. As an incentive, participants were offered the option to be entered into a raffle for a chance to win one of three USD 100 Amazon Gift cards. The Qualtrics platform was used to collect anonymous data. Respondents provided informed consent before beginning the survey questions. Data from this correlational study was analyzed using IBM SPSS V29 with statistical significance set at *p* > 0.05. The survey measures for the two groups were the same with variation in the survey wording according to the level of practice. Similarly, the data was evaluated for the RN group and the NP group individually.

## 4. Results

There were 3911 responses recorded when the survey was conducted. After the data was cleaned and coded, and participants with missing responses removed, 3167 surveys (1934 RN and 1233 NP) were included in the study analysis. Respondents were primarily from a national sample with some international responses. Academic and community settings were represented. In total, 48% of respondents had at least a BSN and 11.7% had a PhD (2.7%) or a DNP (9.0%). Most respondents were women (Table 1), with the largest age group between 25 and 44 years (Table 2), and with the largest group having 1–5 years of nursing practice and a range of 1 year to over 25 years (Table 3). Nurses in this study learned about NM from a variety of resources, including formal courses or workshops, and 30% of respondents were not familiar with NM at all.

Using the predictive value determination of the Mayo Clinic WBI survey (Table 4), 63% of RNs in the study were at high risk of burnout, and 50% of the NPs were at high risk for burnout.

For the RN sample, higher levels of well-being as identified in the Mayo Clinic survey correlated with strong statistical significance for all Narrative Medicine questions in the survey (*p* < 0.001). In addition, the Pearson correlation coefficients demonstrated a weak negative correlation (r= −0.1 to −0.2) between the NM items and the WBI score (Table 5). For the nurse practitioner respondents, higher levels of well-being correlated with moderate-to-strong statistical significance with four NM practice survey questions: (1) the importance of learning the patient’s story, (2) the degree to which the patient’s story increased the NP’s self-understanding, (3) the belief that not getting the patient’s story negatively impacted NP care, and (4) the practice of journaling or other forms of writing about their patient’s stories. The Pearson coefficients for these significant questions ranged from low-to-moderate correlations (r = 0.007 − (−0.6)) (Table 6).

## 5. Limitations

This study has several limitations worthy of discussion. First, as a cross-sectional correlational design, it identifies associations at a single point in time and cannot establish causation or a temporal sequence between Narrative Medicine practices and nurses’ well-being. Second, reliance on self-reported measures introduces the potential for measurement bias. The researcher-developed survey assessed participants’ perceived engagement with NM principles, which may be subject to social desirability bias, whereas respondents overreport behaviors viewed as professionally or ethically desirable. Additionally, there may be recall inaccuracies or differences in individual interpretations of NM. As both the NM practice items and the Mayo Clinic Well-Being Index rely on self-reporting, common method variance may also have contributed to the observed correlations.

Third, the convenience sampling and snowballing methodology is a non-probability sampling method that may introduce selection and affinity bias. Participants may share similar professional networks, workplace environments, and attitudes towards Narrative Medicine. This may over-represent nurses who are more engaged with reflective practices and may have more access to professional networks. These factors may limit the generalizability of the findings. Despite the sample being large and geographically diverse, these biases should be considered when interpreting observed associations between Narrative Medicine practices and well-being.

In addition, the offer of an incentive may have contributed to additional self-selection bias. Incentives are common in survey research to boost response rates. However, they can disproportionately attract participants who are motivated by the reward or different response patterns compared to non-participants. However, the probabilistic and low-expected-value nature of a raffle likely minimized this bias compared to guaranteed compensation, particularly in a professional population.

While the WBI survey has been validated with US nurses, the NM practice survey tool was researcher-developed and validated only with expert review rather than full psychometric validation (e.g., factor analysis and test–retest reliability).

## 6. Discussion and Implications for Further Research

The nursing profession is at a critical juncture, with nurses leaving the profession and national applications and enrollment to nursing programs declining. Nursing burnout is associated with poorer outcomes and reduced safety in patient care and contributes to rising nursing turnover [4,5,6,7]. It has already been shown that the NM activities of journaling, writing prose or poetry, or graphic medicine alone or in a group setting are effective practices [19]. These practices have demonstrated improvement in patient symptoms, emotional health, and quality of life [19]. Furthermore, prioritizing employee well-being, cohesiveness, engagement, and teamwork through human affiliation, collaboration, trust, loyalty, and support has shown to have a positive impact on nurse leaders [20]. It is imperative to pinpoint the causes of workforce challenges, including burnout, and find ways to attract and retain nurses at all levels of practice. This study shows a correlation between better well-being and a nursing/nurse practitioner practice that incorporates key principles of Narrative Medicine. It is important to understand what aspects of Narrative Medicine can be disseminated through nursing education and find ways to incorporate NM into nursing practice. Questions remain, including determining if there is a causative effect of NM and well-being and if improved well-being independently leads to higher retention in nursing. Other questions to answer regarding nursing workforce challenges include examining the differences between nursing practice and nurse practitioner practice regarding NM and well-being. These insights can inform strategies to incorporate NM practice opportunities into the workplace. Further investigation is also needed to identify best practices for integrating NM into nursing curriculums.

## 7. Conclusions

While NM is understood as an effective tool for building empathy and connection and shows positive outcomes for patients and providers, it is essential to understand how NM may contribute to improved well-being and resilience among nurses at all levels of practice. Integrating NM practice approaches into nursing education and professional development could support retention and promote sustainable workforce well-being.

Further inquiry is needed to understand and promote the best methods for teaching NM. For example, results from one quasi-experimental design showed that a Narrative Medicine-based intervention could bring positive effects on the health profession’s students. This study demonstrated that the use of Narrative Medicine to form an empathetic connection could bring positive impacts on health profession students regarding professional identity, self-reflection, emotional catharsis, and self-reflective writing competency [27].

Further research is needed, including using objective or multi-source measures of NM engagement, longitudinal designs, probability sampling, and fully validated instruments to help strengthen causal inferences. This will help us better understand how and when NM is best incorporated into nursing practice and nursing education. It will also help us further understand the impact of NM on nursing providers’ well-being and commitment to remain in the profession. Participants in our study had variable exposure and knowledge about NM. Some learned about it through nursing coursework within other nursing courses, some took stand-alone NM courses, while others learned through professional workshops. None of these have been tested to determine best practices for teaching the necessary skills of NM.

Additionally, identifying opportunities to support NM in a variety of practice environments is critical. At a time when health care costs are rising, it is imperative to consider cost effectiveness and the return on investment when providers have opportunities to connect with their patients and engage in reflective practices. Clinical environments supporting NM through individual and group activities such as journaling and engaging with the arts are areas to be explored and tested.

## Figures and Tables

**Table 1:** Demographics: Gender.

Gender	Percent
Female	73.1%
Male	23.7%
Non-binary/third gender	1.3%
No response	1.4%
Prefer not to say	0.3%
Prefer to self-describe	0.3%

**Table 2:** Demographics: Age.

Age Group	Percent
18–24 years old	12.7%
25–34 years old	45.3%
35–44 years old	28.3%
45–54 years old	8.5%
55–64 years old	3.3%
65+ years old	0.9%
Under 18	0.5%
No response	0.6%

**Table 3:** Demographics: Years of experience.

Years of Experience	Percent
1–5 years	30.3%
6–10 years	32.5%
11–15 years	20.4%
16–20 years	9.1%
21–25 years	3.0%
More than 25 years	4.0%
No response	0.7%

**Table 4:** Predictive value for burnout (higher score indicates worse outcomes).

Outcome	Nurses (RNs)	Advanced Practice Nurses (NPs)
Risk of burnout	4× higher risk	9× higher risk
Risk of severe fatigue	2× higher risk	3× higher risk
Risk of poor overall quality of life	2× higher risk	4× higher risk
Higher risk of recent patient care error	2× higher risk	1.7× higher risk
Risk of moderate or greater intent to leave current position (next 24 months)	2× higher risk	3× higher risk

**Table 5:** Pearson correlations between NM items and well-being (RN sample).

Narrative Medicine RN Survey Item	r	*p*
It is important to understand each patient’s story about their illness and life.	−0.148	<0.001
I always find a way to learn the patient’s story, even when pressured by time or other circumstances.	−0.262	<0.001
Hearing the patient’s story about their illness in their life makes me a better nurse.	−0.206	<0.001
Hearing patients’ stories deepens my understanding of myself.	−0.249	<0.001
Learning the context of illness in my patient’s life helps me be a better nurse.	−0.217	<0.001
When I don’t get the patient’s story, my nursing care suffers.	−0.180	<0.001
Empathy is important in my nursing practice.	−0.091	<0.001
When I learn my patient’s story of their illness, I make a more accurate nursing diagnosis.	−0.247	<0.001
Mindful attention is necessary to learn the patient’s story.	−0.182	<0.001
I often journal, write poetry, or use other forms of writing about my patients’ stories.	−0.296	<0.001
Developing a partnership with my patients improves health outcomes for my patients.	−0.194	<0.001
Developing professional affiliations with other nurses or other professionals makes my nursing practice more rewarding.	−0.211	<0.001

**Table 6:** Pearson correlations between NM items and well-being (NP sample).

Narrative Medicine NP Survey Item	r	*p*
It is important to understand each patient’s story about their illness and life.	−0.10	0.726
I always find a way to learn the patient’s story, even when pressured by time or other circumstances.	−0.60	0.035
Hearing the patient’s story about their illness in their life makes me a better nurse practitioner.	0.015	0.598
Hearing patients’ stories deepens my understanding of myself.	0.007	0.007
Learning the context of illness in my patient’s life helps me be a better nurse practitioner.	−0.010	0.722
When I don’t get the patient’s story, my nurse practitioner care suffers.	0.057	0.047
Empathy is important in my nurse practitioner practice.	0.056	0.051
When I learn my patient’s story of their illness, I make a more accurate diagnosis.	−0.009	0.766
Mindful attention is necessary to learn the patient’s story.	0.010	0.734
I often journal, write poetry, or use other forms of writing about my patients’ stories.	−0.148	<0.001
Developing a partnership with my patients improves health outcomes for my patients.	−0.010	0.729
Developing professional affiliations with other nurse practitioners or other professionals makes my practice more rewarding.	−0.041	0.147

## Data Availability

Data are available from the corresponding author upon reasonable request.

## Supplementary Materials

The following supporting information can be downloaded at: https://www.mdpi.com/article/10.3390/nursrep16010032/s1, Mayo Clinic Well-Being Measurement Surveys for Registered Nurses and Advanced Practice Providers.

## Public Involvement Statement

There was no public involvement in any aspect of this research.

## Guidelines and Standards Statement

This study is reported in accordance with the Strengthening the Reporting of Observational Studies in Epidemiology (STROBE) guidelines.
