# Supplementary material for: The Relationship Between Narrative Medicine and Nurse and Nurse Practitioner Well-Being
Source: Nurs Rep. 2026 Jan 20;16(1):32. doi: 10.3390/nursrep16010032 (PMC12844882; doi:10.3390/nursrep16010032)
Supplement: Supplementary file 1 [file nursrep-16-00032-s001.zip › nursrep-4051256-supplementary.pdf]

Supplementary Materials

Mayo Clinic Well-Being Measurement Surveys for Registered Nurses and Advanced Practice Providers

Nurse Well-Being Index

During the past month...

have you felt burned out from your work? Y/N

have you worried that your work is hardening you emotionally? Y/N

have you often been bothered by feeling down, depressed, or hopeless? Y/N

have you fallen asleep while sitting inactive in a public place? Y/N

have you felt that all the things you had to do were piling up so high that you could not overcome them? Y/N

have you been bothered by emotional problems (such as feeling anxious, depressed, or irritable)? Y/N

has your physical health interfered with your ability to do your daily work at home and/or away

from home? Y/N

Please rate how much you agree with the following statements

The work I do is meaningful to me

7-point Likert scale; anchor “very strongly disagree” at the 1 end of the scale and “very strongly agree” at the 7 end of the scale

My work schedule leaves me enough time for my personal/family life

strongly agree; agree; neutral; disagree; strongly disagree (State of Well-Being 2022-2023, 2023)

Advanced Practice Providers Well-Being Index

During the past month...

have you felt burned out from your work? Y/N

have you worried that your work is hardening you emotionally? Y/N

have you often been bothered by feeling down, depressed, or hopeless? Y/N

have you fallen asleep while sitting inactive in a public place? Y/N

have you felt that all the things you had to do were piling up so high that you could not overcome them? Y/N

have you been bothered by emotional problems (such as feeling anxious, depressed, or irritable)? Y/N

has your physical health interfered with your ability to do your daily work at home and/or away from home? Y/N

Please rate how much you agree with the following statements

The work I do is meaningful to me

7-point Likert scale; anchor “very strongly disagree” at the 1 end of the scale and “very strongly agree” at the 7 end of the scale

My work schedule leaves me enough time for my personal/family life strongly agree; agree; neutral; disagree; strongly disagree (State of Well-Being 2022-2023, 2023)
